# “We do others’ Jobs”: a qualitative study of non-nursing task challenges and proposed solutions

**DOI:** 10.1186/s12912-024-02059-9

**Published:** 2024-07-15

**Authors:** Hekmat Al-Akash, Ayman Aldarawsheh, Rami Elshatarat, Murad Sawalha, Ahmad Saifan, Nezam Al-Nsair, Zyad Saleh, Wesam Almagharbeh, Dena Sobeh, Mudathir Eltayeb

**Affiliations:** 1 Nursing Department, Faculty of Nursing, Applied Science Private University, Amman, 11937 Jordan; 2 Intensive Critical Care Unit, Royal medical services, Prince Rashid military hospital, Irbid, 21110 Jordan; 3 Department of Medical and Surgical Nursing, College of Nursing, Taibah University, Madinah, 42353 Saudi Arabia; 4 Department of maternal, child and family health nursing, Faculty of Nursing, The Hashemite University, Zarqa, 13133 Jordan; 5 College of Nursing, Xavier University, Cincinnati, OH 45207 United States of America; 6 Department of Clinical Nursing, School of Nursing, The University of Jordan, Amman, 11942 Jordan; 7 Nursing Department, Vision Colleges, Riyadh, 13226 Saudi Arabia; 8 Medical Surgical Nursing Department, Faculty of Nursing, University of Tabuk, Tabuk, 47512 Saudi Arabia; 9 Department of Medical Surgical Nursing, College of Nursing, Prince Sattam bin Abdulaziz University, AlKharj, 16278 Saudi Arabia

**Keywords:** Non-nursing tasks, Jordanian nurses, Qualitative research, Healthcare workload, Role ambiguity, Task assignment

## Abstract

**Introduction:**

Non-nursing tasks (NNTs) have become a prevalent issue among healthcare professionals, affecting nurses globally. This study delves into the experiences of Jordanian nurses regarding NNTs, aiming to uncover challenges and propose solutions within the Jordanian healthcare context.

**Objective:**

Explore the impact of NNTs on Jordanian nurses’ roles, workload, and satisfaction. Additionally, the study aims to identify various types of NNTs performed by nurses, understand their impact, and propose solutions to mitigate challenges associated with these tasks.

**Methods:**

A qualitative-exploratory research design was employed for this study. Semi-structured interviews were conducted with Jordanian nurses using a purposeful sampling approach to ensure a diverse representation of experiences and perspectives. Thematic analysis was used to identify recurring themes and patterns related to NNTs, their challenges, and potential solutions. Ethical guidelines were strictly followed to maintain participant confidentiality and ensure the integrity of the data collected.

**Results:**

Analysis of the interviews revealed four major themes: challenges of NNTs, types of NNTs, impact of NNTs, and proposed solutions. Nurses faced significant difficulties due to task ambiguity, role confusion, and increased workload from NNTs, which included administrative duties, clerical work, and tasks typically performed by other healthcare professionals. These NNTs negatively impacted nurses’ effectiveness, productivity, and job satisfaction by diverting time and energy from primary nursing responsibilities, causing professional strain. To address these issues, participants suggested clearer job descriptions, stricter task assignment protocols, and systemic changes to tackle the root causes of NNTs.

**Conclusion:**

This study sheds light on the pervasive challenges posed by NNTs among Jordanian nurses and emphasizes the importance of addressing these issues to enhance nursing care quality and nurse well-being. By proposing actionable solutions tailored to the Jordanian context, this research contributes to the global discourse on NNTs and underscores the need for organizational support and advocacy to optimize nurses’ roles and improve patient care outcomes.

## Introduction

Nurses play a critical role in healthcare, providing direct patient care, advocating for patients’ well-being, and contributing to a positive healing environment. However, their ability to fulfill these core responsibilities can be hampered by a phenomenon often referred to as “non-nursing tasks” (NNTs) [1–3]. These tasks, as the title “We Do Others’ Jobs” suggests, can encompass a wide range of duties that fall outside the traditional scope of nursing practice. Examples might include administrative work, answering phones, or even cleaning duties.

In Jordan, the typical nursing job description encompasses a wide range of responsibilities, including providing direct patient care, administering medications, monitoring patient progress, coordinating with other healthcare professionals, and educating patients and their families about health maintenance and disease prevention. Nurses are expected to uphold high standards of clinical practice and adhere to strict ethical guidelines while managing their patients’ overall health and well-being. However, despite these defined roles, Jordanian nurses frequently encounter non-nursing tasks (NNTs) that detract from their primary responsibilities. These tasks often include administrative duties, clerical work, and activities typically performed by other healthcare staff. There is a significant gap in knowledge regarding the prevalence and impact of NNTs on nurses, both within Jordan and in the international context. This study aims to address this gap by exploring the challenges posed by NNTs and their consequences on nurses’ professional lives, thereby contributing to a better understanding of how to enhance nursing practice and patient care.

Nurses often find themselves diverted from their core caregiving duties, assuming various non-nursing roles due to moral obligation and organizational pressures [2, 4, 5]. Increased awareness of nursing identity, spurred by academic education, has led nurses to vocalize concerns about this issue [5]. Redesigning organizational processes and fostering inter-professional cooperation through policy changes are suggested to address this challenge [2, 4]. Practical solutions include structuring work environments to minimize nurses’ NNTs by allocating efficient support services, thus reducing undone nursing tasks [6]. Nursing students’ perception of the profession can be improved by enhancing their preparedness through orientation programs [1]. Nurse managers should monitor activities and reallocate efforts to prioritize nursing care, thereby reducing missed care and enhancing client outcomes [7]. Systemically, continuous support for nursing identity and redesigning care models to encourage cooperation among healthcare professionals are essential [8]. Healthcare education frameworks should instill moral obligations universally to foster future inter-professional partnerships [2, 4]. Additionally, rethinking staff compositions and skill mixes is necessary to adapt to evolving healthcare demands [5].

Technology offers potential in reducing NNTs by automating administrative tasks through healthcare information systems, though successful adoption in Jordan requires infrastructure and resource considerations [9]. Inter-professional collaboration, promoting task distribution based on expertise, aligns with global trends toward team-based care models, potentially easing NNT burdens [10]. Re-evaluating staffing and considering additional support roles can alleviate pressure on nurses, optimizing workforce efficiency [11, 12].

In the US, nursing services encompass various roles, with registered nurses assuming responsibilities from physical examinations to research, while nurses in low-middle-income countries face challenges like job ambiguity and work overload [12–14]. Jordanian nurses experience exacerbated role ambiguity due to NNT performance, highlighting the need for contextual solutions [1]. Understanding these differences is crucial for tailoring strategies to local contexts and effectively addressing NNT challenges.

The prevalence of NNTs in nursing practice has gained global attention, including in Jordan, where concerns about patient care quality and nurse well-being have escalated [1]. Clarifying nursing roles is crucial to prevent NNT encroachment and ensure efficient task allocation [15]. However, existing research on NNTs in Arab nations, particularly Jordan, remains limited, with a focus on quantitative studies and a lack of exploration into interventions and solutions [1, 16, 17]. Qualitative research is essential for understanding the complex contextual factors contributing to NNTs and devising targeted interventions [5, 18].

While NNTs are not a new issue in healthcare, their prevalence and impact on nurses, particularly in developing countries, warrant further exploration. In Jordan, nurses are primarily responsible for direct patient care, including administering medications, monitoring vital signs, providing wound care, and educating patients and their families. Additionally, they coordinate care, manage patient records, and ensure overall patient well-being. However, Jordanian nurses often find themselves burdened with NNTs such as administrative duties, clerical work, and tasks that should be performed by other healthcare professionals, which detract from their primary responsibilities [19, 20]. This qualitative study delves into the experiences of Jordanian nurses with NNTs to fill the knowledge gap about this topic both nationally and internationally. Through in-depth focus group interviews, we aim to understand the specific challenges Jordanian nurses face due to NNTs, the impact on their workload and well-being, and their perspectives on potential solutions. Our research highlights the significant strain that NNTs place on nurses, leading to role confusion, increased workload, and decreased job satisfaction [21]. By exploring these themes, this study seeks to contribute to the growing body of research on NNTs and advocate for targeted solutions that allow nurses to concentrate on their core competencies. Ultimately, addressing the issue of NNTs can lead to improved patient care, better job satisfaction for nurses, and a more efficient healthcare system.

## Study objectives

The current study aims to fill this gap by exploring Jordanian nurses’ perspectives and experiences regarding NNTs, seeking strategies to mitigate their occurrence in the local healthcare context. This qualitative approach is crucial for gaining nuanced insights and developing effective interventions tailored to Jordan’s specific needs [22].

The specific objectives of this study are as follows: (1) To explore the nuanced experiences and perspectives of Jordanian nurses regarding NNTs within the Jordanian healthcare context; (2) To identify and classify the various types of NNTs that Jordanian nurses encounter in their daily practice, including administrative duties, patient transport, and other tasks traditionally performed by non-nursing personnel; (3) To examine the impact of NNTs on nurses’ roles, workload, job satisfaction, and overall well-being in Jordanian hospitals; (4) To investigate the root causes of NNTs within the Jordanian healthcare system and understand their far-reaching consequences for nurses and patients; and (5) To propose potential solutions and interventions to mitigate the challenges posed by NNTs and improve the quality of nursing care and nurses’ working conditions in Jordanian hospitals.

## Methodology

### Design

The objective of this research was to delve into nurses’ perspectives on NNTs in Jordan, aiming to comprehend and classify these tasks based on nurses’ viewpoints [23, 24]. The decision to employ a qualitative exploratory design stemmed from the lack of clear definitions of NNTs in the Jordanian context and the novelty of investigating NNTs in Jordan [25, 26]. A qualitative approach was chosen for its capacity to gather detailed, nuanced information and provide a thorough understanding of the social phenomena under scrutiny [23, 27]. This methodology excels in addressing questions of “why” and “how,” aligning well with the objective of exploring varied perceptions and interpretations of NNTs across different healthcare settings in Jordan.

### Settings

Jordan’s healthcare system is organized into four principal sectors: public, private, university, and military, a delineation established by Al-Hamdan et al. (2017) [28]. This study targeted three expansive hospitals to investigate the phenomenon of NNTs, each representing a different sector: governmental, educational, and private institutions. The selection process for these hospitals was meticulous, focusing on their strategic placement in Amman, which is the urban hub where approximately 40% of Jordan’s population resides. This strategic location facilitated access to a broad spectrum of nursing professionals and provided insights into the challenges faced by healthcare workers in the capital.

Furthermore, these hospitals were chosen to ensure a diverse sample of nurses from various regions across Jordan. The chosen governmental hospital represents the public sector and offers a wide range of services to a large patient population. The educational hospital, associated with a major university, not only provides healthcare services but also serves as a training ground for future healthcare professionals, representing the university sector. The private hospital, representing the private sector, caters to patients who seek specialized and often more immediate care.

These hospitals boast multidisciplinary departments and a substantial nursing workforce, factors that eased the process of data collection and enriched the study’s findings. By encompassing these diverse contexts, the study aimed to glean a nuanced understanding of NNTs among nurses in Jordan. This comprehensive approach ensured that the findings reflected the varied experiences and challenges of nurses across different healthcare sectors and geographic regions, providing a holistic view of the impact of NNTs on nursing practice in Jordan.

### Population and sample

This study aimed to understand how registered nurses (RNs) experience and perceive NNTs in their daily work. To gain valuable insights, the research specifically recruited nurses with recent and direct patient care experience. Purposive sampling, a method focusing on specific qualities, ensured participants had the relevant knowledge. Nurses included were actively involved in providing care across various hospital departments within the past six months. Those solely in managerial positions or not directly involved in patient care, like research or education, were excluded.

The qualitative nature of the research, seeking in-depth understanding, meant a smaller sample size was ideal. This allowed for focused discussions where each nurse could share their unique perspective on NNTs. Focus group interviews were chosen as the best way to collect data. These groups, with 4 to 10 participants each, fostered an environment where diverse viewpoints could be exchanged. Rich discussions within the groups would generate a deeper understanding of NNTs’ impact on nurses.

To ensure a well-rounded picture, the study recruited a total of 38 experienced nurses from three different hospitals. This strategic selection encompassed nurses from various departments within each hospital, reflecting the diverse realities of nursing practice. By including participants from different settings and departments, the research aimed to capture a broad spectrum of experiences with NNTs. This ultimately enriched the data by providing a more nuanced understanding of how nurses perceive and deal with NNTs in their daily work. Overall, this sampling strategy targeted a specific group of experienced nurses with diverse backgrounds, allowing the research to explore the complexities of NNTs from a variety of perspectives within the nursing profession.

### Data collection method

For data collection, this research employed focus group interviews, a method specifically chosen to leverage the power of group interaction. These sessions aimed to not only gather individual perspectives on NNTs but also to spark collaboration and discussion among participants. This collaborative environment fostered a richer exchange of ideas, potentially leading to the emergence of unique viewpoints or shared challenges that might not have surfaced in individual interviews.

The focus groups were conducted in carefully chosen spaces. Privacy was paramount, ensuring participants felt comfortable expressing their honest opinions and experiences with NNTs. The chosen locations were also conducive to open and engaging discussions, free from distractions. To ensure a comprehensive record of the sessions, all focus groups were audio-recorded with the participants’ consent. These recordings were then supplemented with detailed notes taken by the facilitator throughout the discussions. These notes captured not only the verbal content but also any non-verbal cues or body language that could provide additional insights.

The interview format chosen for the focus groups was semi-structured. This approach utilized a pre-defined interview protocol, outlined in Table 1, to guide the discussion and ensure all key areas of interest related to NNTs within the Jordanian healthcare context were covered. The protocol included a series of open-ended questions designed to prompt participants to reflect on their understanding of NNTs and their impact on their work. These questions also encouraged participants to share their personal experiences, both positive and negative, related to NNTs. Additionally, the protocol incorporated questions that solicited suggestions for mitigating the prevalence of NNTs and potentially streamlining workflows within Jordanian healthcare settings. The semi-structured nature of the interview format allowed for flexibility. The facilitator could probe deeper into interesting points raised by participants or explore related topics that emerged organically during the discussion. This flexibility ensured that the focus groups captured not just the pre-determined areas of interest but also the full range of nurses’ experiences and perspectives on NNTs.

**Table 1:** Interview Guide (Schedule)

1. **How do you define the concept of NNTs?** 2. **What do NNTs mean from your point of view?** 3. **How can we decrease the spread of NNTs within Jordanian health settings?**	

### Ethical considerations

Ethical clearance was secured from the nursing ethical approval for human research committee of Institutional Review Board (IRB) at Applied Science Private University the Applied Science Private University (#2022-NC-119) and the respective hospitals involved in this study, safeguarding the rights and well-being of participants. All methods were conducted in strict adherence to the relevant guidelines and regulations, including the principles outlined in the Declaration of Helsinki [32].

Participation in the research was entirely voluntary, with no coercion or repercussions for declining involvement. Prior to their participation, each potential participant received a detailed invitation letter delineating the study’s objectives and procedures. Informed consent, indicating an understanding of the study’s purpose and procedures, was obtained from all participants, who retained the right to withdraw from the study at any point without consequence. To ensure the safety and comfort of participants, interviews were conducted in secure, public settings, guaranteeing confidentiality and minimizing any potential discomfort or coercion. Furthermore, to protect participants’ identities and uphold confidentiality, pseudonyms were employed throughout the study, thereby anonymizing their contributions and ensuring privacy. These ethical measures were rigorously implemented to uphold the principles of respect, autonomy, confidentiality, and beneficence throughout the research process.

### Data Analysis

Thematic analysis, following the framework outlined by Braun and Clarke (2019), was employed to scrutinize the interview data [29]. This method entails several phases, including coding techniques to identify and annotate underlying concepts, organizing related material into groups, and linking disparate concepts and themes. The process comprises six steps for data extraction [29].

To commence the analysis, the writers familiarized themselves with the data by listening to the taped interviews multiple times. This step aimed to distill the essence of the experiences shared by participants. The interviews were then meticulously transcribed verbatim in Arabic, the language spoken by the participants, to ensure the authenticity and accuracy of the narratives. Subsequently, the transcribed data were transferred to a computer for further examination.

During the analysis, data were coded and labeled based on the similarity of concepts, facilitating the grouping of information. This process generated around 100 free nodes, formed through the aggregation of codes into categories, subthemes, and overarching themes, each reflecting shared or unique meanings.

To ensure rigor and consistency in the analysis, consensus on the interpretation of units, codes, categories, and themes was sought among the researchers. This collaborative approach facilitated inter-coder agreement, enhancing the credibility and reliability of the findings. Additionally, the NVivo program was utilized to manage and organize the data, aiding in the systematic and comprehensive analysis of the thematic patterns emerging from the interviews.

## Results

### Description of the study sample

The study sample consisted of 38 skilled and dedicated nurses selected from three prominent Jordanian hospitals, with six focus groups facilitating comprehensive data collection—two groups had seven participants each, while four groups had six participants each. Careful selection criteria ensured a diverse representation of the nursing workforce, capturing various perspectives and experiences related to NNTs. The sample included slightly more male nurses (57.9%) than female nurses (42.1%). Participants’ ages ranged from 24 to 48 years, providing insights into how different age groups perceive and manage NNTs. Nurses’ experience ranged from 1 to 24 years, with an average of 9.4 years. Approximately one-third of participants worked in government (34.2%) and educational (34.2%) sectors, while the rest were in private healthcare institutions (31.6%). The study included nurses from various departments, with the majority in medical-surgical wards (38.4%) and intensive care units (31.6%), ensuring that diverse experiences and perspectives were captured across different specialties and care settings (Table 2).

**Table 2:** Demographic characteristics of participants

Variable	Mean (SD) or *n* (%)
**Gender**	
Male	22 (57.9%)
Female	16 (42.1%)
**Age (year)**	32.1 (5.9)
**Health Sector**	
Government	13 (34.2%)
Education	13 (34.2%)
Private	12 (31.6%)
**Years of Experience (year)**	9.4 (3.7)
**Department**	
Intensive care unit	12 (31.6%)
Medical-surgical ward	26 (38.4%)

### Study themes and sub-themes regarding NNTs in Jordanian hospitals

A qualitative study examined Jordanian nurses’ experiences with NNTs. Interviews revealed four key themes (Table 3): (1) Challenges of NNTs, (2) Types of NNTs, (3) Impact of NNTs, and (4) Proposed Solutions. Challenges included unclear roles and increased workload due to NNTs, potentially harming patient care. The types of NNTs were wide-ranging, encompassing administrative duties, patient transport, tasks typically done by doctors, and even those of other healthcare professionals. Engaging in NNTs negatively impacted nurses’ effectiveness and job satisfaction. Finally, nurses proposed solutions like clear task assignments, improved staffing, well-defined job descriptions, better communication, and advocating for their rights. Understanding these challenges and solutions is crucial for optimizing nurses’ roles and ensuring quality patient care.

**Table 3:** Study Themes and Sub-Themes regarding NNTs in Jordanian Hospitals

Theme	Sub-Themes	Description	Example Quotes
**Challenges of NNTs**	- Task ambiguity and role confusion - Nurse workload and staffing shortages - Impact on patient care	Nurses perform tasks beyond their job descriptions due to unclear roles and staffing shortages, affecting both nurses’ well-being and patient care quality.	- “NNTs are those tasks that are not in our job descriptions…” (Nurse 21) - “We are already working more than our capacity.” (Nurse 10)
**Types of NNTs**	- Clerk and management tasks - Transporting tasks - Physician tasks - Tasks of other healthcare professionals	Nurses perform various NNTs, including administrative duties, transporting patients and samples, tasks typically done by physicians, and tasks of other healthcare professionals.	- “We do the clerk’s duties.” (Nurse 29) - “Nurses carry added worry as we handle tasks like taking patients to radiology…” (Nurse 11) - “I am doing my job in addition to physician jobs…” (Nurse 37) - “Nurses do others’ jobs. We do chest physiotherapy…” (Nurse 26)
**Impact of NNTs**	- Reduced effectiveness and productivity - Nurse demotivation and career dissatisfaction	Engaging in NNTs can decrease nurses’ effectiveness, hinder professional growth, and lead to demotivation, potentially impacting their commitment to the profession.	- “…may divert nurses from opportunities for ongoing education…”
**Proposed Solutions**	- Task assignment and accountability - Staffing and resource allocation - Clear job descriptions and adherence - Resolving confusion and uncertainty - Building nurses’ personalities and activating nursing unions	Nurses proposed various solutions to address NNTs, including clear task assignments, adequate staffing, well-defined job descriptions, clear communication channels, building assertiveness in nurses, and advocating for their rights through nursing unions.	- “Creating a task map that details who is responsible for what tasks…” (Nurse 6) - “Increasing nursing staff and allocating adequate resources…” (Nurse 10) - “Imagine a hospital where each nurse’s job description is crystal clear…” (Nurse 13) - “This can be resolved by transferring the situation in the department…” (Nurse 26) - “Building nurses’ personalities during their university studies would be a solution.” (Nurse 2)

### Challenges of NNTs in Jordanian hospitals

Numerous interviewees expressed frustration regarding the allocation of tasks beyond their official job descriptions. One nurse succinctly stated:

*“NNTs are those tasks that are not in our job descriptions, but we are still expected to do them” (Nurse 21).*

This highlights the crucial necessity for clarity in defining nurses’ roles to mitigate ambiguity and alleviate additional stress. Additionally, nurses emphasized that NNTs frequently arise due to shortages of licensed staff, with some nurses assuming the duties of unlicensed personnel. This added workload compounds existing pressures in hospitals, as articulated by Nurse 10:

*“NNTs are any tasks that are not related to nursing and are assigned to the registered nurse due to a shortage of unlicensed nursing staff. We are already working more than our capacity.”*

### Types of NNTs

While some nurses acknowledged the potential learning opportunities and professional enrichment associated with engaging in NNTs, the majority emphasized the importance of striking a balance between NNTs and primary patient care responsibilities to prevent adverse effects on nurses’ concentration and patients’ health. Analysis of the interviews revealed five subthemes, including clerk and management tasks, transporting tasks, and physician tasks, among others. Numerous nurses identified certain administrative and clerk duties as examples of NNTs. For instance, many nurses mentioned their responsibility for continually checking patients’ files to ensure they contain all necessary documents or signed consent forms. Administrative duties encompass extensive documentation, patient chart management, and inventory management.

*“We are responsible for managing everything for patients. This includes managing their admission and discharge. We do the clerk’s duties.” (Nurse 29)*.

Amidst their myriad responsibilities, nurses shed light on additional aspects of their workload, expressing concerns about tasks such as transporting patients to radiology and obtaining medications from the pharmacy. Moreover, many nurses revealed that they often spend significant time transporting blood, urine, and other samples from their departments to the laboratories, highlighting challenges with the availability and responsiveness of transporters.

*“Nurses carry added worry as we handle tasks like taking patients to radiology and getting medications from the pharmacy.” (Nurse 11)*.

The interviews unveiled widespread discussion among participants regarding task overlaps between nurses and physicians, with variations observed across different hospitals. Notably, the governmental hospital exhibited more pronounced overlap compared to the other two hospitals. Participants highlighted instances where specific physician tasks, such as inserting peripheral intravenous lines, blood sampling, nasogastric tube insertion, and changing surgical and wound dressings, became integrated into nurses’ responsibilities.

*“I am doing my job in addition to physician jobs in my hospital. I insert intravenous lines and withdraw blood samples.” (Nurse 37)*.

In five out of six interviews, more than half of the nurses discussed numerous NNTs performed by nurses, including tasks such as medication preparation, such as diluting and preparing chemotherapy. While acknowledging overlaps with other health professionals’ tasks, nurses emphasized that these professionals should receive support from nurses rather than solely relying on them to complete these tasks. Some interviews revealed instances where nurses assumed responsibilities typically attributed to physiotherapists and respiratory therapists, particularly in critical care departments. Additionally, nurses frequently took charge of inquiring about patients’ dietary preferences and checking for prescribed or special diets in patient files.

*“Nurses do others’ jobs. We do chest physiotherapy before and after giving bronchodilators, and we also help patients with exercise. These are the tasks of physiotherapists.” (Nurse 26)*.

### Impact of NNTs

Engaging in NNTs can have adverse effects on a nurse’s effectiveness, productivity, and professional growth. These responsibilities may divert nurses from opportunities for ongoing education and career development, hindering their ability to fully grasp clinical intricacies and make informed decisions. The burden of NNTs may also lead to internal conflicts and demotivation among nurses, potentially raising concerns about their long-term commitment to the nursing profession. Hence, it was imperative to inquire about potential interventions and solutions from nurses to address this social phenomenon and mitigate its impact on both nurses and the quality of healthcare services.

### Proposed solutions

Proposed solutions emerged as a multifaceted theme during the in-depth interviews, encompassing several subthemes. These ranged from establishing clear task assignments and accountability mechanisms to addressing ambiguity surrounding nurses’ roles. Participants also emphasized the importance of nurturing nurses’ professional identities during their academic training and mobilizing nursing unions to champion nurses’ rights. Through these proposed solutions, the theme advocates for comprehensive strategies aimed at streamlining task management, bolstering the nursing workforce, and empowering nurses to prioritize their core responsibilities. Ultimately, these measures aim to uphold high standards of patient care and cultivate a supportive and conducive work environment within healthcare settings.

#### Task assignment and accountability

Participants from different hospitals emphasized the importance of specific task assignments and individual accountability as potential solutions to address the challenge of NNTs. They highlighted the significant role of management in task allocation, suggesting that strategic mapping of tasks could streamline healthcare operations. One nurse articulated this idea, stating,

*“Creating a task map that details who is responsible for what tasks can facilitate smoother coordination. For instance, a designated nurse for medication administration reduces duplication and improves accuracy” (Nurse 6).*

Transparency in task distribution was also seen as crucial by nurses, as it empowers them and fosters accountability. They recommended implementing a feedback loop that holds individuals accountable for task outcomes. A nurse emphasized this approach, saying,

*“Publishing a clear task roster detailing each nurse’s assignments promotes a sense of ownership. When everyone knows their responsibilities, we can collectively ensure that no essential tasks fall through the cracks” (Nurse 13).*

In the pursuit of effective solutions, nurses explored innovative approaches to promote equity and skill exchange. One such approach was a rotation-based task assignment system, aimed at enhancing fairness and collaborative skill-sharing among nursing professionals. Nurses supporting this strategy highlighted its potential to optimize task distribution and knowledge exchange. According to one nurse,

*“Rotating tasks among nurses ensures a fair distribution and encourages cross-sharing of skills. Nurses can learn from each other’s strengths, and it prevents one nurse from being overburdened with the same task repeatedly” (Nurse 8, 18).*

Granting nurses autonomy and empowerment through task choice emerged as another compelling solution. Nurses advocated for allowing nurses to select tasks they feel confident and competent in handling, believing that it could boost morale and enhance accountability. One nurse explained,

*“Empowering nurses to choose tasks they feel competent and comfortable with can boost morale and accountability. When nurses have a say in their assignments, they are more invested in the outcome” (Nurse 17).*

#### Staffing and resource allocation

Participants in the study emphasized the critical importance of addressing staffing shortages and allocating sufficient resources to mitigate the challenges posed by NNTs. Nurses [1, 28, 34] highlighted the significance of hiring additional staff to address the existing shortage and increasing nursing staff while allocating adequate resources to alleviate the burden of NNTs. Echoing a similar sentiment, Nurse [10] stated,

*“Increasing nursing staff and allocating adequate resources would alleviate the burden of NNTs.”*

Some nurses proposed a visionary solution to addressing NNTs, focusing on breaking down traditional silos between disciplines within healthcare and fostering interdisciplinary collaboration. Nurse [22] articulated this perspective, saying,

*“Breaking silos and fostering collaboration across disciplines is an advanced strategy to address NNTs. Imagine a scenario where non-clinical tasks are handled by dedicated personnel, allowing nurses to focus exclusively on patient care. This interdisciplinary synergy optimizes skill utilization and enhances the patient experience.”*

In the ever-evolving landscape of nursing practice, innovative solutions have emerged to mitigate the challenges posed by NNTs. One such solution involves augmenting the workforce by increasing the number of aid nurses, porters, and support personnel. Nurses [11, 13, 33] envisioned a collaborative environment where dedicated aides manage specific tasks, enabling nurses to concentrate on core patient care responsibilities. They stated,

*“Increasing the presence of aid nurses, porters, and support staff to handle specific tasks marks a monumental step towards alleviating the impact of NNTs. Imagine a collaborative environment where dedicated aides expertly manage tasks such as patient transportation, enabling nurses to concentrate on intricate medical interventions. This comprehensive approach does not just ease the burden; it harmonizes the entire healthcare team’s efforts, enhancing patient comfort, expediting processes, and elevating the standard of care we deliver.” - Nurses* [11, 13, 33].

#### Clear job descriptions and adherence

Participants emphasized the importance of clear job descriptions and strict adherence to them as essential measures to reduce confusion and ambiguity surrounding NNTs. Nurses highlighted the significance of this solution, emphasizing the need to follow precise job descriptions to prevent the assignment of tasks unrelated to nursing roles.

*“Imagine a hospital where each nurse’s job description is crystal clear—like a roadmap guiding us. When job roles are spelled out and strictly followed, there is less room for NNTs to sneak in. For instance, think about patient assessments. With a clear job description for this task, nurses can focus on giving patients the attention they need, without being diverted by unrelated duties.” - Nurse* [13].

The collective wisdom of nurses drives us towards a ground-breaking solution that finds widespread support: creating new job descriptions. Nursing professionals echo the transformative potential of this strategy, recognizing the need for a fresh perspective on roles and responsibilities in addressing the complexities of NNTs.

*“Imagine if our job descriptions were like tailor-made suits, precisely fitted to our skills, knowledge, and the evolving demands of healthcare. A collective effort to create new job descriptions would involve each nurse contributing their insights, painting a vivid picture of their roles. This customization would ensure that each nurse’s strengths are magnified, leading to optimized patient care.” - Nurses* [8, 10].

*“Dynamic healthcare requires fluidity in job descriptions. With most nurses advocating for new descriptions, a panorama of possibilities unfolds. Job descriptions should reflect the multifaceted nature of our roles, allowing for adaptation and specialization. This evolutionary approach would enable us to navigate NNTs with agility and ensure that patient care remains at the forefront.” – Nurses* [12, 15, 18, 20, 24].

#### Resolving confusion and uncertainty

Resolving confusion and uncertainty around nurses’ duties and responsibilities emerged as a key solution to address NNTs, as proposed by participants. Nurses advocated for seeking resolution by involving higher authorities, emphasizing the importance of clear communication channels and engagement with hospital leadership.

*“This can be resolved by transferring the situation in the department to the hospital director for resolution, such as activating the roles of orderlies and patient companions. Imagine if there is a direct line to the hospital director, like a safety net. When nurses face a task that seems out of place, they can raise it to the director for review. For instance, if a nurse is repeatedly asked to handle equipment repairs, they can escalate the situation, prompting the hospital to ensure the proper maintenance staff is involved.” - Nurse* [26].

Similarly, nurses underscored the transformative potential of elevating the issue to higher administrative levels, envisioning a scenario where hospital leadership reshapes task assignments to prioritize nurses’ core responsibilities.

*“Taking it up a level can be the game-changer. If nurses encounter tasks like transportation that disrupt their core duties, they can bring it to the hospital director’s attention. Picture a nurse highlighting how this affects patient care and the director stepping in to allocate resources for dedicated staff, like patient companions, to manage these responsibilities.” - Nurse* [8].

Moreover, nurses emphasized the importance of clear communication channels to address confusion and uncertainty, envisioning a hospital environment where information flows seamlessly, enabling nurses to focus on their core responsibilities.

*“Addressing the confusion and uncertainty around nurses’ duties and responsibilities through clear communication channels would be beneficial. Imagine a hospital where information flows like a well-tended garden. When everyone knows what is expected of them, including NNTs, it is like sunshine breaking through clouds. For example, if a nurse understands exactly when and how to collaborate with administrative staff, it minimizes the chances of being saddled with unrelated tasks.” - Nurse* [10].

#### Building nurses’ personalities and activating nursing unions

Participants also proposed building nurses’ personalities during university studies and activating nursing unions to address NNTs. Nurses highlighted the potential of building nurses’ personalities during their academic journey, stating,

*“Building nurses’ personalities during their university studies would be a solution.” - Nurse* [2].

As nurses navigate the landscape of NNTs, a transformative approach involves building the personalities of nurses and activating nursing unions. Nurses proposed that empowering nurses with assertiveness and self-advocacy can be pivotal.

*“Empowering nurses to assert themselves and advocate for their professional roles is the cornerstone. When nurses face NNTs that hinder patient care, they can stand up and voice their concerns to the management. This reinforces the nurse’s sense of professional identity and encourages the establishment of dedicated personnel for non-clinical tasks.” - Nurse* [5].

Amidst the challenges posed by NNTs, nurses recognize the pivotal role that the Jordanian Nurses and Midwives Council can play in advocating for their rights and providing essential support. Nurses proposed that the council’s intervention could be a game-changer in this scenario.

*“Calling upon the Jordanian Nurses and Midwives Council is a strategic move. As the guardian of our professional standards, the council can advocate for clear job descriptions and allocate resources to alleviate NNTs. Imagine nurses approaching the council with data on how these tasks affect patient outcomes, and the council mobilizing efforts to ensure our expertise is channeled towards direct patient care.” - Nurse* [8].

In addressing NNTs, nurses are increasingly vocal about their rights and values within the healthcare system. A prevalent sentiment among nurses is the importance of advocating for fair compensation when asked to perform tasks outside their core responsibilities. Most nurses have united in this call, recognizing the significance of their contribution and the need for just acknowledgment.

*“Taking a stand for our rights is not just about protesting; it is about demanding equitable recognition. Nurses are beginning to unite to make management acknowledge our extra workload. Imagine a scenario where nurses join forces and collectively ask for compensation, reflecting the time and effort invested in these tasks. We are not just requesting payment; we are advocating for our worth. If management can see that our role is not limited to our job descriptions but extends to these additional tasks, they should compensate us fairly, acknowledging the value we bring to the healthcare team.” - Nurse* [19].

## Discussion

The investigation into Jordanian nurses and NNTs: challenges and solutions provides a comprehensive examination of how NNTs impact the roles, workload, and satisfaction of nurses in Jordan. Employing a qualitative research approach, this study delved deeply into the nuanced experiences and perspectives of Jordanian nurses regarding NNTs. Through semi-structured interviews and thematic analysis, the intricate layers of the NNT phenomenon within the Jordanian healthcare context were explored. The journey into this realm revealed a complex landscape of challenges and opportunities, drawing from the narratives and insights shared by Jordanian nurses, unearthing a wealth of information on the identification, classification, and impact of NNTs. The findings of this study align with global trends, as Jordanian nurses, similar to their international counterparts, are engaged in various NNTs, including administrative work and patient transport. These findings corroborate prevalent NNTs reported in diverse healthcare settings globally [2, 6, 30].

This study holds several unique distinctions, notably being the inaugural qualitative investigation into nurses’ perceptions of NNTs, a term with varied definitions globally [5]. Moreover, it is imperative to acknowledge the broad spectrum of tasks considered as NNTs, including activities such as phone answering and administrative duties, which may not be universally classified as such [31, 32]. Furthermore, most related studies have been conducted in developed nations, underscoring the significance of this research in the context of a developing country like Jordan [5, 30, 33].

Nurses articulated their frustration with tasks that extended beyond their official roles, aligning with previous research findings [1, 5]. These tasks, ranging from administrative duties to patient transport responsibilities, not only impeded their effectiveness but also hindered their career advancement prospects. These findings resonate with earlier studies highlighting the detrimental effects of NNTs on job satisfaction, morale, and ultimately, patient care, as observed in the previous researches [6, 17, 28]. Additionally, nurses expressed concerns about unclear role definitions, which contributed to further confusion in task allocation.

In response to these challenges, participants proposed various solutions, including clearer job descriptions, stricter task assignment protocols, and increased involvement of higher authorities. Moreover, building nurses’ assertiveness and self-advocacy, along with activating nursing unions, emerged as potential strategies to address these issues. These proposed solutions align with prior research emphasizing the crucial role of organizational support, managerial interventions, and advocacy efforts, as noted in the previous study [6].

The investigation among nurses in Jordan illuminated the state of NNTs in this particular setting, consistent with global patterns seen in other studies. Like their international counterparts, Jordanian nurses often engage in a variety of NNTs, including administrative work, answering phones, obtaining supplies, cleaning medical equipment and patient rooms, and patient transportation [5, 30, 31]. These findings align with prevalent NNTs reported in various healthcare contexts globally [30, 34].

Addressing the issue of NNTs is crucial for improving nursing care quality and nurse well-being. The current study aligns with existing literature while offering unique insights into proposed solutions, emphasizing the importance of clear task assignments, accountability mechanisms, and well-defined job descriptions [6]. Adequate staffing and resource allocation are critical strategies for mitigating the burden of NNTs, resonating with suggestions from previous research [6].

Confusion and uncertainty around nursing duties present challenges that need to be addressed through early education on task delineation, especially during clinical training, as recommended in the previous study [1]. Additionally, the development of nurses’ assertiveness and the activation of nursing unions play crucial roles in advocating for nurses’ rights and influencing healthcare policies, aligning with prior research findings [8].

Advocacy, fair compensation, and recognition of nurses’ contributions are essential components of the solution, motivating nurses and acknowledging their critical role in patient care [35]. Further qualitative research is warranted to better understand the contextual factors and consequences of NNTs experienced by nursing staff and students [36]. Additionally, the development of instruments measuring the complexity of NNTs is crucial for comprehensive assessment, as highlighted by Grosso et al. (2019) [5]. This study contributes valuable insights from a Jordanian context, emphasizing the importance of tailoring solutions to specific cultural and contextual factors.

Addressing NNTs is crucial for improving nursing care quality and nurse well-being. The study underscores the importance of clear task assignments, accountability mechanisms, and well-defined job descriptions to prevent NNTs from encroaching on nurses’ workloads. Strategies such as adequate staffing and resource allocation are deemed critical for mitigating the burden of NNTs, necessitating system-level changes to address underlying resource constraints. Moreover, efforts to clarify nursing duties and distinguish them from tasks assigned to other healthcare providers are essential. Nursing students’ expressions of concern regarding these distinctions underscore the need for early education on task delineation to prevent confusion in the future workforce. Furthermore, developing nurses’ assertiveness and activating nursing unions emerged as pivotal strategies in advocating for nurses’ rights and influencing healthcare policies. Encouraging nurses to assert their identity and rights aligns with recommendations for continuous support to strengthen nursing identity, as highlighted in previous studies.

The findings of this study resonate with previous research on NNTs and their impact on nurses, particularly in terms of the challenges and negative consequences associated with these tasks. Similar to studies conducted in Western countries [5, 37, 38], our research identified task ambiguity, role confusion, and increased workload as significant challenges faced by nurses when performing NNTs. These issues were consistently highlighted by our participants, who reported that the ambiguity surrounding their roles often led to inefficiencies and reduced job satisfaction. Furthermore, like the findings of previous studies [1, 5], our study noted that engaging in NNTs significantly detracted from the time and energy nurses could devote to direct patient care, thus impairing the quality of care provided. Moreover, our study expands on the existing literature by providing insights into the specific types of NNTs encountered by nurses in Jordan and their unique impacts within this regional context. While the types of NNTs identified, such as administrative duties and clerical work, align with those found in studies from other regions ^6,16,21,30^, our research highlights the compounded effects of NNTs in a resource-limited healthcare system. The diverse sample from various hospital settings in Jordan revealed that nurses often bear additional burdens due to systemic inefficiencies and staff shortages, which are more pronounced compared to some Western healthcare systems [30, 31, 38]. This study thus contributes to the global understanding of NNTs by demonstrating that, while the nature of these tasks and their impacts are universally challenging, the specific context of a country’s healthcare infrastructure can exacerbate these issues, calling for tailored interventions that address both local and universal aspects of nursing practice.

Overall, while this study offers valuable insights into the challenges and potential solutions regarding NNTs among Jordanian nurses, further research, particularly qualitative investigations, is warranted to fully understand the contextual factors and consequences of NNTs for nurses globally. Advocacy, fair compensation, and recognition are essential for motivating nurses and acknowledging their critical role in patient care, underscoring the need for continued efforts in this area.

### Research implications and recommendations

The examination of NNTs within the nursing profession reveals a multifaceted challenge requiring a comprehensive approach for resolution. Research findings, including those from Uneken (2018), underscore the critical link between NNTs and patient care quality [39]. Increased NNTs correlate with decreased perceived care quality and a rise in missed care, highlighting the direct impact on patient outcomes when nurses are burdened with tasks beyond their core responsibilities. Solutions proposed in the literature, such as advocacy for fair compensation and recognition of nurses’ contributions, emphasize the need for organizational change prioritizing patient-centered care over NNTs. Purohit and Vasava (2017) suggest restructuring care models to enable inter-professional collaboration, ultimately alleviating NNT burdens by assigning tasks more efficiently [8].

For nurse managers, understanding the root causes of NNTs within the Jordanian healthcare system provides insights to develop evidence-based strategies for alleviation. Insights from studies by Grosso et al. (2019) and Ayasreh et al. (2022) inform nurse managers in creating more efficient work environments and optimizing nursing resources to improve care quality [1, 5]. By addressing NNT challenges proactively, nurse managers can enhance operational efficiency and care quality, ultimately benefiting both nurses and patients [16, 38].

Policy makers and decision-makers in the healthcare sector can leverage the findings of this research to guide the development of policies and the allocation of resources, effectively addressing the prevalence and impact of NNTs in Jordan. By aligning new policies with empirical evidence, they can optimize the use of nursing resources, thereby enhancing patient outcomes [7, 22]. This evidence-based approach allows healthcare institutions to implement targeted reforms that reduce the burden of NNTs on nurses. Such reforms could lead to more patient-centered care, stronger safety protocols, and increased operational efficiency [40, 41]. As healthcare systems evolve in response to these findings, patients can expect a higher standard of care, with nurses able to focus more on direct patient care, resulting in better overall experiences and outcomes. The reduction of NNTs not only improves job satisfaction and productivity among nurses but also ensures that healthcare delivery is more efficient and effective, ultimately benefiting the entire healthcare system [3].

### Research limitations

One potential limitation of this study is the reliance on self-reporting through interviews, which may introduce bias or inaccuracies in participants’ responses. Additionally, the study’s focus on Jordanian nurses may limit the generalizability of findings to nurses in other cultural or healthcare contexts. Furthermore, the qualitative nature of the study may restrict the ability to quantify the prevalence or severity of NNTs experienced by nurses. Moreover, the study’s cross-sectional design may capture only a snapshot of participants’ experiences and may not fully capture changes over time. Lastly, the sample size and recruitment method could affect the representativeness of the findings, as it may not encompass the full spectrum of experiences among Jordanian nurses.

## Conclusion

The study comprehensively examined NNTs experienced by Jordanian nurses, revealing significant challenges and proposing solutions. It found that NNTs, ranging from administrative duties to tasks outside their official roles, strain nurses’ workload and compromise patient care. Organizational interventions such as clearer job descriptions and adequate staffing are crucial to address NNT challenges effectively.

The study highlights the importance of organizational interventions to mitigate the burden of NNTs. Clearer job descriptions and enhanced communication channels are proposed solutions to alleviate challenges associated with NNTs. Additionally, adequate staffing and resource allocation are essential for reducing the frequency of NNTs and optimizing nursing roles, ensuring better patient care delivery. Moreover, the study emphasizes the role of nurse advocacy and recognition in influencing healthcare policies. By advocating for their rights and asserting their identity, nurses can contribute to improving patient care quality and enhancing their professional satisfaction. Overall, the study provides valuable insights for policymakers, nurse managers, and healthcare organizations to optimize nursing roles and foster a supportive work environment for Jordanian nurses.

## Data Availability

Data availabilityThe datasets used and/or analysed during the current study are available from the corresponding author on reasonable request.
